# The fast and the furious—An experimental investigation of the pace of life and risky speed choice in traffic

**DOI:** 10.1371/journal.pone.0236589

**Published:** 2020-07-27

**Authors:** Carina Goldbach, Christin Hoffmann, Julia Hoppe, Thomas Pitz, Kirsten Thommes

**Affiliations:** 1 Hochschule Rhein-Waal, Kleve, Germany; 2 Brandenburg University of Technology Cottbus-Senftenberg, Cottbus, Germany; 3 Organizational Behavior, Paderborn University, Paderborn, Germany; Shandong University of Science and Technology, CHINA

## Abstract

Despite discernible improvements in the last decades, speeding is still a pertinent problem for road safety, fuel efficiency, and greenhouse gas mitigation. In order to understand individual speeding decisions, we need a better understanding of who speeds. In our paper, we test whether individuals' general pace of life is associated with speeding decisions. We use a novel speed-choice experiment that confronts participants with a scenario in which they repeatedly decide between driving fast or slow. This decision is associated with different accident risks. Before the experiment, each participant's pace of life was measured. Our results show that individuals with a slower pace of life are more likely to choose slow in the experiment and are also more likely to switch to slow, even when they had success by driving fast in the preliminary round. Therefore, individuals' pace of life may contribute to our understanding of speeding.

## Introduction

Two types of garter snakes occupy the boundaries of the behavioral continuum: At the one end, some snakes move fast but are less likely to fully exploit their territory, as they are less likely to leave once habituated paths. On the other end of the spectrum, there are rather slow snakes that eventually discover more of their territory [1]. The explanatory factor differentiating fast and slow snakes is their individual pace of life. Without knowing that the pace-of-life syndrome will have been later found valid in behavioral biology, researchers in transportation psychology assumed already in 1978 that there are similar types of humans. The individual perceptions of time may explain human behavior in traffic to a great extent [2]. The pace-of-life syndrome in animals is currently in the spotlight of research attention. It roots back to the classical fast-slow life-history continuum, assuming that between and within species-variation in physical attributes like metabolism and hormones is causal for variation in behavior [3]. Risk attitudes are assumed to be related to the physical pace of life. Some animal species' high pace of life is associated with high risk-taking and less thorough exploration [4]. In this paper, we set out to analyze empirically whether the pace of life can also contribute to our understanding of human speeding behavior in traffic, as speeding behavior is still one of the main road safety problems. According to the United States Department of Transportation [5], speeding caused 9,378 deaths in 2018 in the US. Even though many drivers agree that speeding is not worth the risk, many of these drivers also speed, at least occasionally [6]. This speeding-paradox explains why attitudes towards speeding are poor predictors for speeding choices [7].

Contrary to attitudes about the right speed choice, past research found situational characteristics and personality to be more relevant predictors of speeding [8,9]. Situational factors such as distance traveled or purpose of driving [10] apply to every driver and the between driver heterogeneity in speeding choice, mainly explained by personality and socio-demographic factors. Previous research shows that speeding differences are associated with both surface-level differences such as gender or age and deep-level differences, for example, risk attitude and sensation seeking [9,11–15]. Begg and Langley [16], for example, found that the tendency to speed is more prevalent in males and age-dependent. Attitudes, values, and beliefs more easily define some speeders than by demographic categories [9,17].

Next to the relatively frequently mentioned factors such as age, gender, and risk, individual personal perceptions of time have been discussed and are currently becoming more relevant in analyzing speeding decisions. According to the early work of Stokols et al. [2], individual perceptions of impedance matter for speed choices: Having traveled relatively longer distances at a relatively slow speed leads to higher perceptions of congestion and an increased likelihood of speeding. One central result of Jorgensen and Polak [18] is that the individual’s attitude towards travel time savings appears essential. Current research on commuting [19] takes up this observation, highlighting that individual impedance is a very personal assessment of time. We contribute to this literature by analyzing the association between the individual pace of life and speeding: Psychological research [20,21] transfers biological research on differences between and within species to human behavior. That research assumes that individuals experience a given time very differently and distinguishes between high-paced and slow-paced individuals. The pace of life is assumed to be a personal trait and may have a strong influence on human behavior, within which is the decision to drive fast or slow.

In our paper, we theoretically explain why time preferences may impact speeding choice and add to the current literature by introducing the pace of life as an essential personal explanatory factor. We test our model empirically by measuring an individual's pace of life and relating this measure to risky choices in an economic experiment. In this economic experiment, participants are asked to decide about risky speed choices in traffic over 100 consecutive rounds. Our results reveal that individuals' pace of life explains not only speeding decisions but also their inclinations to change their driving strategy.

### Pace of life and risky choices

An essential aspect discussed in the literature on perceptions of time is the concept of the pace of life [20,21]. According to the socio-emotional selectivity theory, individuals differ in their ability to assess time and arrange different frames and periods [22]. Levine and Norenzayan [21] use the "pace of life" to capture this varying perception of time in different cultures. They approximate the pace of life in a region by measuring mean walking speed, mean working speed, and mean clock accuracy: They find remarkable differences between cities and countries on the aggregate level. As a follow-up, economists find some evidence that an aggregated higher pace of life in a society is associated with better collective economic outcomes such as economic prosperity [23]. This claim can be supported by the fact that Western Europe has the fastest pace of life [21].

Moreover, researchers find higher punctuality and walking speed in economically developed countries such as the United States or Japan compared to developing countries [24,25]. Although some studies confirm the relationship between the pace of life and economic success, there is little research on the micro basis of these results. However, it is necessary to explore this link to understand the impact and effect of the pace of life on human behavior.

In contrast with human behavior research, the concept of the pace of life is well researched in biology concerning different animal species behaviors. In biological research, the pace of life is an established concept. Researchers find remarkable results on the micro-level, which could help understand the impact of the pace of life. This literature deals with explaining how life-history traits can explain individual variation in behavior within and between animal species. As a result, research shows that life history differences correlate with animals' individual behavior [1].

Some researchers establish an organismal biological approach by claiming that life-history characteristics, together with physiological traits in environmental conditions and evolutionary history, are forming a pace-of-life syndrome. Biological research assumes that differences in the pace of life can be traced to differences in ecological environments and natural adaptation. This pace of life is associated with differences in behavior between and within species according to (1) risky behavior, (2) speed of movement, and (3) habituation of behavior.

Unlike in the animal kingdom, the pace-of-life syndrome approach has received only minor attention in human behavior literature. Del Giudice [26] highlights that the fast-slow continuum can be a productive heuristic for individual differences, but also points out the field should update its theoretical assumptions, e.g., explore new approaches in light of the human ecology. Međedović [27] argues that the behavioral ecological approach offers theoretical concepts that provide clear hypotheses that possibly can also be tested on humans, but that this task is not easy to transfer from animal to human research. Nevertheless, he recommends that researchers apply the evolutionary ecology approach to the psychology of human personality.

In the biological research, there is an association between the pace of life and risky behavior: When comparing amphibians, reptiles, fish, and other species with indeterminate growth, Stamps [28] finds that species with a higher pace of life in terms of growth rates are more willing to take risks, for example in foraging, than slow-growing animals would. Hall et al. [29] support these results in their research on birds. They additionally find that more exploratory birds have a lower survival rate, which matches the pace-of-life syndrome hypothesis that individuals who have a short life are more risk-prone. Huntingford et al. [30] find that carps who display high-risk behavior have a higher resting metabolic rate than carps who take lower risks. In this stream of literature, the pace of life is measured as the longevity of species. Short-lived entities are here found to be more risk-prone and proactive than long-lived individuals. Hence, life-history strategies (slow and fast) and risky behavior can be connected [3].

Réale et al. [3] show that individuals who mature faster, first exhibit increased risk behavior, and second have higher scores on cardiovascular parameters than individuals who mature more slowly. If physiology and risk behavior are mediators of the trade-off between current and future reproduction in a similar way, we can also assume a positive correlation between risk behavior and cardiovascular parameters. Moreover, Lehmann et al. [31] analyze adolescent girls regarding their health-related risk behavior and find that fast-maturing adolescents have overall higher blood pressure and express more risk-taking behavior than slow-maturing girls and boys of the same age. Therefore, we assume:

*H1*: *There is a positive correlation between an individual’s pace of life and risk attitude*.

There is also an association between the pace of life and the level of activity in biological research. Careau et al. [32] explore whether dogs with a faster pace of life measured by high rates of growth, mortality, and energy expenditure share other characteristics such as boldness, aggression, and high activity levels. Their results support the existence of a pace-of-life syndrome resulting from the coevolution of metabolic, personality, and life-history traits. Similarly, Gangloff et al. [1] investigate fast and slow living in snake behavior regarding the pace of life and life-history strategies. They focus on tongue flicks as behavior and movement speed as a pace of life measure, strongly correlated. Fast living types are found to be more active.

Snakes' pace of life is also associated with variability in behavior and the tendency to explore new habitats. Faster paced-snakes are here less-likely to change routines, displaying signs of habituation. These snakes tend to be more likely to choose similar areas in their territory even though they were generally more active. Gangloff et al. [1] conclude that fast living organisms use higher activity and information-gathering levels to pave the way for higher efficiency than their slow living counterparts.

To sum up, biological research shows that a fast pace of life is associated with high metabolic rates [33] and the behavioral tendency to be a proactive personality [1,3,32]. If these results can be applied to speeding behavior in humans, we would expect that a faster pace of life is associated with a higher inclination to speed. We hypothesize:

*H2*: *The higher an individual's pace of life*, *the more often the individual chooses to drive fast in the speeding experiment*.

Research on snakes [1] shows that pace of life is also surprisingly correlated with the inclination of habituation. Thus, faster-paced individuals are less likely to explore opportunities compared to slower-paced snakes of the same species. Transferred to human behavior, we would expect fewer changes in strategies but rather an increased inclination to choose the same strategy repeatedly. We hypothesize:

*H3*: *The higher an individual's pace of life*, *the higher the individual's probability of retaining a chosen speeding strategy*.

To test the three hypotheses above, this research paper combines a measure of the pace of life with actual repeated speeding choices. A major difficulty in the analysis of day-to-day traffic choices is obtaining repeated observations of actual behavior. Therefore, most studies focusing on individual speeding choices rely on self-reports or official speed measurements in traffic [34]. Both types of studies have disadvantages: self-reported data is restricted to individual perception and might suffer from social-desirability biases or recall biases (see e.g., [35]). Speed data obtained in actual traffic, on the other hand, usually leads to only one observation per driver and limited additional information (see, e.g., [18]). Hence, both methods are limited to take individual variability into account and link it to the individual pace of life measures. To overcome this difficulty, laboratory experimental approaches, in which real persons are involved in a simulated repeated traffic situation, are conducted. These kinds of experiments have generally provided a basis that serves as a bridge between theoretical development on the one side and full-scale field studies on the other [36].

Thus, the present study aims to address these shortcomings by investigating repeated speeding behavior and linking to the pace of life in a controlled laboratory setting, as game-theoretic experiments have already been successfully used in other areas of traffic research. Especially notable are the many studies on commuters' route choice that investigate in a very similar setting the participants' choice for one of two routes (see, e.g., [37–40]). In these route choice experiments, payoffs depend on the time needed to go from A to B and assumes that driving times can be represented by a monetary payoff function. The time needed from A to B again depends on the number of participants choosing the respective route – the more people chose one route, the more congestion occurs, the more time is needed to arrive, and thus the lower the payoff. While the payoff depends on the other participants' behavior, there is no risk included, and everyone reaches the destination. We decide to follow these laboratory studies on traffic choices and represent driving times by monetary payoff functions. Unlike the experiments described above, we included risk as a particular characteristic of speeding in this game-theoretic setup. Thus, we design a speed choice experiment in which participants have to decide whether to drive fast or slow and link these choices to individual measures of the pace of life.

## Methods

We draw on the extensive game-theoretic literature on route choices and conduct a novel speed choice experiment to test our hypotheses on the link between the individual pace of life and speeding decisions.

### Ethical statement

The laboratory experiment was conducted at the Rhine-Waal University of Applied Sciences, which had no ethical committee at the time. Therefore, we followed general ethical standards for conducting laboratory experiments: (1) Participation was voluntary; (2) Participants signed an informed consent about the following aspects: Their participation was voluntary. Everyone had the right to withdraw at any time without any penalty. Participants also signed that their data would be recorded, stored, and analyzed by the University only for research purposes. Anonymity was preserved by assigning the participants with a randomly generated code that cannot be associated with names or student IDs. Every participant knew that s/he was taking part in this study, and no data were collected without consent; (3) Only students participated. Thus, every participant was older than 18 years (full legal age in Germany), and students could write and read at the time of participation. No vulnerable individuals in any other way were involved. (4) This was not a clinical or invasive study, as well as no collection of biological samples, and no health risk to any of the participants was involved. We did not use deception. (5) Methods and procedures were fully transparent to students; incentives were made transparent. Everybody received a show-up fee and a positive amount; participants were at no risk of losing their (own) money. Each participant signed a receipt of his/her payment in the end. (6) After the experiment, students were debriefed via email, and we revealed that their working and walking speed was recorded. Students were given the opportunity to opt-out of the experiment. We asked students for their consent to use the data after students were paid for their participation to ensure that students felt free to opt-out.

### Participants

For this study, 47 students from the Rhine-Waal University of Applied Science, Germany, were recruited through the University's internal database XPERTISE (Experimental Research and Training in Social and Economic Sciences). Students from all study programs could sign up on a first-come, first-serve basis. Their ages ranged from 18 to 35 years. On average, participants earned 59.511 experimental currency units (ECU) (= 4.17€) in the experiment. For answering a consecutive questionnaire, participants received another 5€.

### Experimental design

We use a recently designed novel speed choice experiment in which the participants are organized into groups of eight and instructed to choose between driving either fast or slow in order to get from A to B. The participants' payoff depends on the time needed to arrive in B. If they arrive in B after choosing slow, they received 1 ECU. If participants arrive in B after choosing fast, they receive a higher payoff of 2 ECU. However, every participant faces the risk of having an accident that resulted in not arriving in B and therefore having a payoff of 0 ECU.

**Fig 1:**
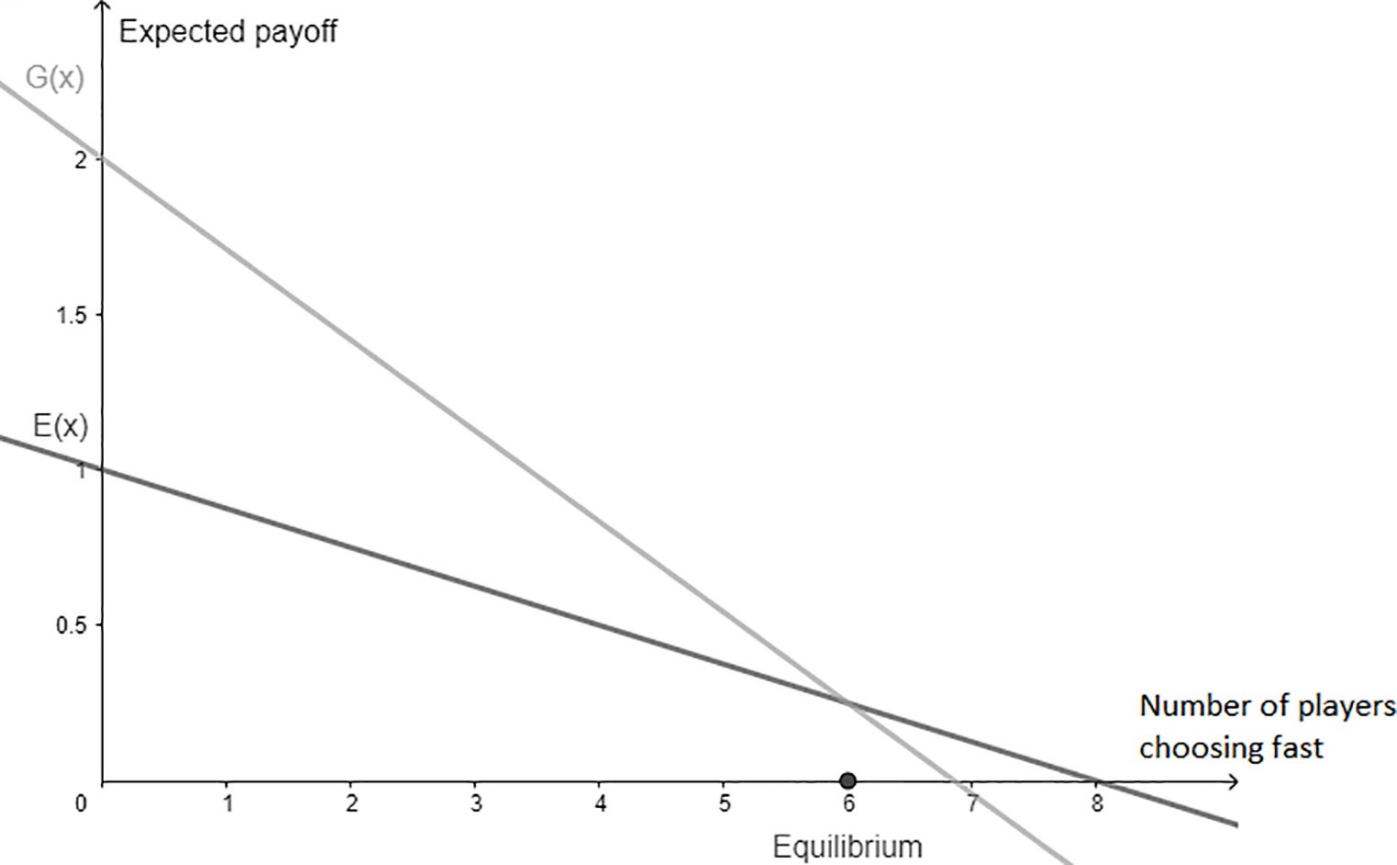
Expected payoff of choosing fast, G(x), and of choosing slow, E(x), depending on the number of players (x) in a group that chose fast.

The experiment is designed to emphasize that the individual's likelihood to experience an accident increases for all participants with the number of fast drivers, but is at any given point higher for fast drivers than for slow ones. Thus, the main characteristic of the underlying model is that an individual's probability to arrive is described by *P_f_*(*x*) if choosing fast and *P_s_*(*x*) if choosing slow:

$$P_{f}(x)=\frac{1}{f}\left(\frac{1}{\frac{-n}{m}}x+f\right)$$ (1)

$$P_{s}(x)=\frac{1}{s}\left(\frac{1}{-n}x+s\right)$$ (2)

where *x* is the number of fast players, *n* the group size, *f* the payoff if arriving after driving fast, *s* the payoff if arriving after driving slow and *m* a parameter for accident risk when speeding (Please note: We use the term speeding as a synonym for choosing fast in the experiment). The equilibrium is reached if the expected payoff of driving fast *G*(*x*) = *fP_f_*(*x*) is equal to the expected payoff of driving slow *E*(*x*) = *sP_s_*(*x*) and solves for *x* = 6. The social optimum, i.e., the highest expected payoff for the group as a whole, is reached when all participants choose *slow*. Fig 1 presents the expected payoff for both strategies, depending on the number of players choosing fast.

This experiment involves coordination in asymmetric outcomes. Coordination games like this have been typically played over a small number of periods. As it has been shown that games with 100 rounds or more produce different behavior and as traffic situations are best described as frequently repeating traveling choices, this experiment runs over 100 identical rounds (see [40]). The total payoff per participant is the sum of all 100 period payoffs with a fixed exchange rate of 0.07 € per ECU.

All participants faced the same scenario. This scenario was not represented graphically in order to avoid framing effects. After each of the 100 rounds participants received information about (1) the number of the current period, (2) their choice in the preceding period, (3) information whether they reached the destination in the preceding period, (4) their period payoff in the preceding period in ECU and (5) their cumulative payoffs in ECU. Complete instructions and a screen-shot of the experiment can be found in the appendix (S1 and S2 Appendices).

### Procedure

At the beginning of the session, all participants were welcomed in a meeting room, handed their participation ID, and individually asked to walk to the next building, in which the laboratory is located, and show their ID to a student assistant. They were instructed to walk directly and to use the covered passageway that connects both buildings. Their walking time was recorded and averaged at around 30 seconds. After all, participants arrived in the computer laboratory; they dropped all their personal belongings, including smartphones and watches outside of the private computer booths, and then chose a PC. They entered their participation ID, and the computer session was initiated. Communication with other participants was not possible during the whole computer session as students were sitting in empty and separated computer booths. In the first task, they were asked to copy the informed consent on a prepared sheet of paper, sign it, and hand it in at the end of the session. Afterward, the speed choice experiment was played for 100 rounds. The computer session concluded with a questionnaire. The time they needed to write the informed consent and their later actions in the experiment and survey were automatically recorded by the experimental software. In total, sessions took around 45 minutes.

### Dependent variables

The dependent variable *fast* shows the choice that participants make by choosing fast driving (1) or slow driving (0) in the experiment (Table 1). *Fast* can be regarded as a risk measure in a traffic context. The cumulative dependent variable of *fast* shows the cumulative number of driving rounds fast. The dependent variable *strategy change* indicates whether the player changes the strategy from either *fast* to *slow* or vice versa (player ends a sequence of one or more equal choices, and switches to the opposite strategy =1, does not switch =0). As each of the 47 participants (=47 independent observations) decided in 100 consecutive rounds, we observe 4,700 total decisions (Table 1).

**Table 1:** Description of dependent variables.

Dependent variables	Percentage	Min	Max	Mean	Std. dev.	N
Risk measure in a traffic context		0	1	0.524	0.499	4,700
Fast (1)	52.43					
Slow (0)	47.57					
Accumulated number of fast rounds per individual		0	100	26.686	19.467	47
Number of strategy changes		0	60s	15.21	11.194	47

### Explanatory variables

In order to answer our research hypotheses, we include the individual's pace of life as an essential personality factor as an explanatory variable. The individual's pace of life is measured in accordance with Levine and Norenzayan [21]. As explained in section 3.3, we have several measures for each participant: (1) a measure of individual walking speed for a consistent walking distance of 39.5 m, (2) a measure of individual working speed when fulfilling a writing task, (3) a measure of their speed in answering a questionnaire and finally (4), a measure of their decision time, when choosing fast or slow. Table 2 gives an overview of the single measures.

**Table 2:** Measures for pace of life.

	Mean	SD	Scoring coefficients	Measurement (in seconds)
Walking time	29.298	8.985	0.510	Not incentivized walking speed over 39.5 meters
Writing time	294.000	91.527	0.238	Time needed for filling in a standard form (approx. 95 words)
Survey time	396.851	118.474	0.490	Time needed for answering 74 questions in the questionnaire
Decision time	4.686	1.288	0.294	Time needed for deciding on fast or slow in the experiment (average over 100 rounds)

Using a confirmatory factor analysis, an ultimate measure for each individual's pace of life was derived by the regression method. We standardized the pace of life measure afterward to the interval [0,1] via z-transformation. To assess the quality of our pace of life measures, we estimate a structural estimation model as one might suspect that our measures show multi-collinearity. We find all measures to significantly contributing to the pace of life measure, with writing time having the best predictive power. The overall goodness of fit is R=.618 and cannot be further improved by removing variables.

We have additional information on age, gender, self-stated risk aversion, self-stated patience, and the possession of a driver's license from the consecutive questionnaire. Risk was measured by "Are you generally a person who is willing to take risks, or do you try to avoid taking risks?” Patience was measured by “Are you generally an impatient person or someone who always shows great patience?” Risk and patience have been evaluated on 0 to 10 scale and were standardized afterward to the interval [0,1] via z-transformation. Additionally, experimental factors like the group's behavior in the past or the experience of an accident in the previous round are expected to affect the participant's speed decision directly. Therefore, they will be taken into account in the subsequent analysis (Table 3). A correlation table for the variables can be found in S3 Appendix.

**Table 3:** Summary statistics of independent variables.

Independent variables	Percentage	Min	Max	Mean	Std. dev.	N
The pace of life [0,1]		0	1	0.647	0.196	47
Accident		0	1	0.571	0.495	4,700
Yes (1)	57.11					
No (0)	42.89					
Risk [0,1]		0	1	0.484	0.247	47
Patience [0,1]		0	1	0.492	0.274	47
Age		18	35	23.979	3.492	47
Gender		1	2	1.532	0.499	47
Female (1)	46.809					
Male (2)	53.191					
License		1	2	1.574	0.494	47
Yes (1)	42.553					
No (2)	57.447					

## Results

The results highlight three crucial aspects of the experiment. First, there is a detailed analysis of the choice of driving slow or fast. In each round, participants in groups of eight had to choose between the slow and the higher rewarding but a riskier fast option. Second, we are highlighting the sum of speed choices that the participant made in the experiment. Last, we analyze the strategy changes that the individuals have made in the 100 rounds of the experiment.

### Descriptive results

**Fig 2:**
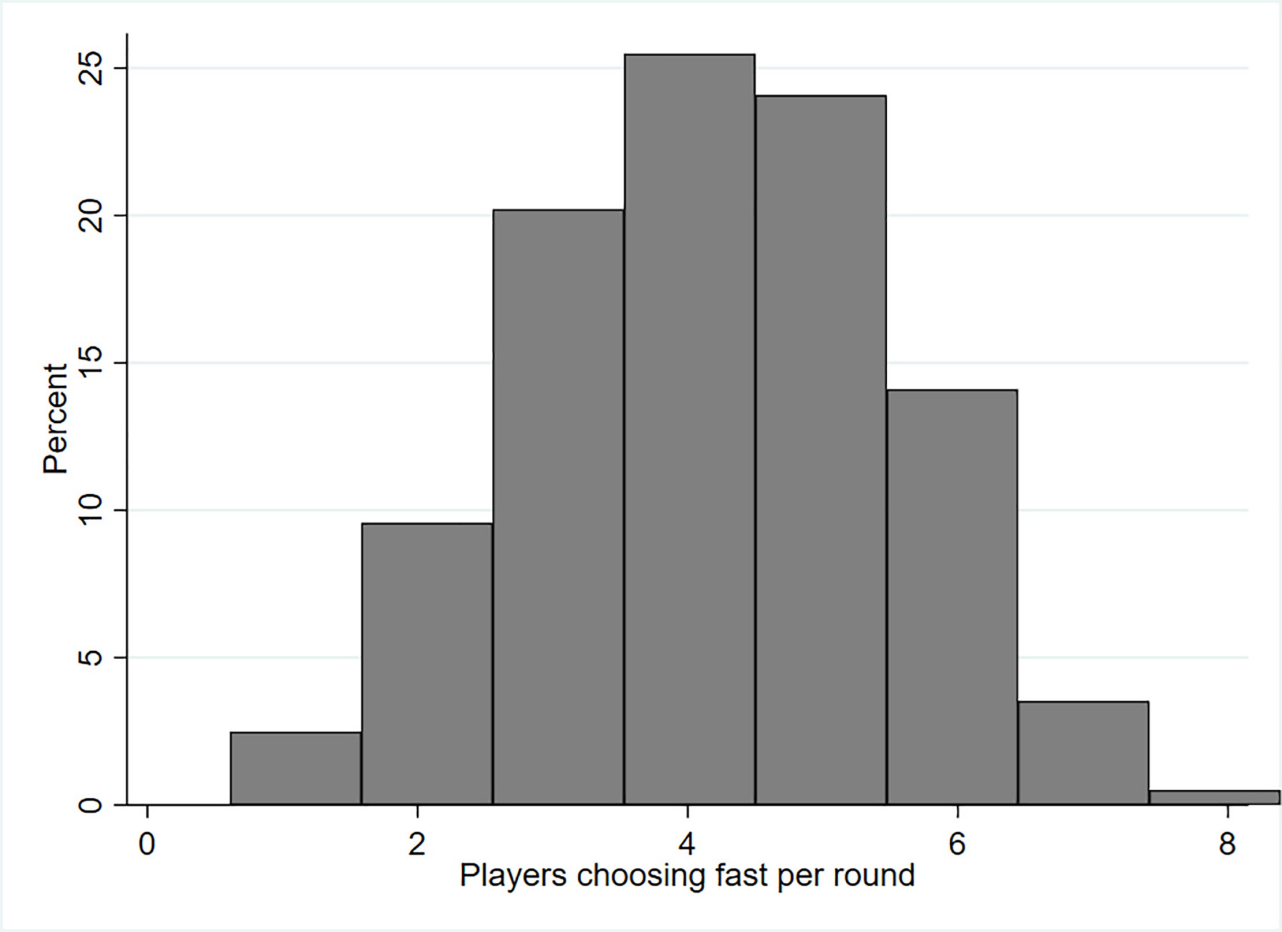
Number of players choosing fast per round in percentage.

The social optimum is reached if all drivers choose *slow*. However, there was no round, in which all players decided to drive *slow* (see Fig 2). Furthermore, there was only one participant who chose fast in all 100 rounds, and one who chose slow in all rounds. On average, 4.18 players decided to drive *fast* (median=4) per round, resulting in an average of 4.66 accidents. Chosen strategies resulted in an average payoff of 59.511 ECU (sd. 14.961). Fig 2 shows that four out of the eight participants chose fast in more than 25% of the rounds played.

**Fig 3:**
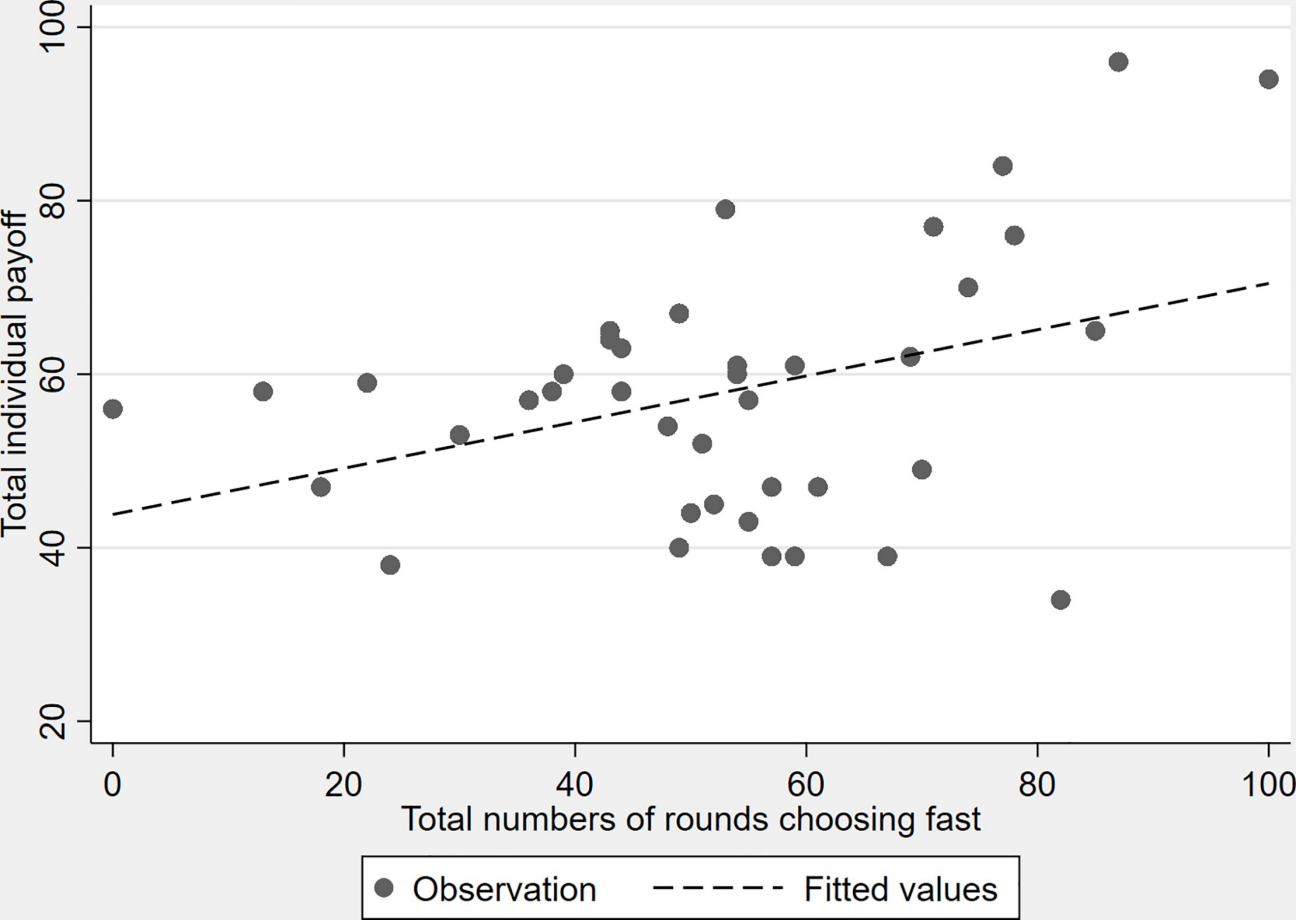
Total payoff depending on the total number of rounds choosing fast.

In Fig 3, it can be seen that the participants' total individual payoff is higher the more frequently a driver has chosen fast; thus, the riskier one's choice was in our traffic context. This holds true for the decision of fast from approximately 40 times upwards.

**Fig 4:**
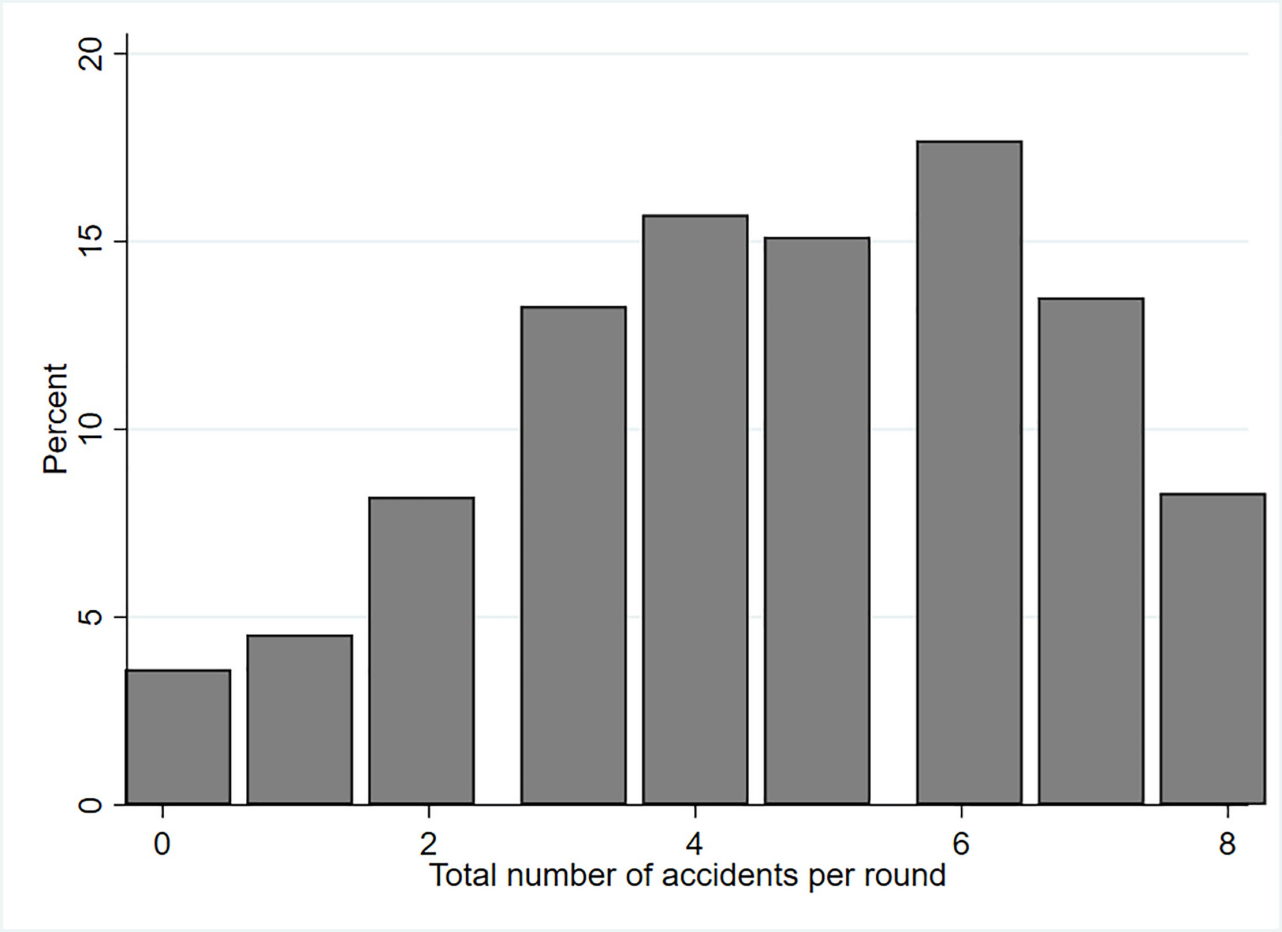
Total number of accidents per round.

The relatively high number of drivers choosing fast (Fig 4) also results in a relatively high number of drivers involved in an accident and therefore do not reach B, resulting in zero payoff in the respective round.

### Pace of life and risk

**Fig 5:**
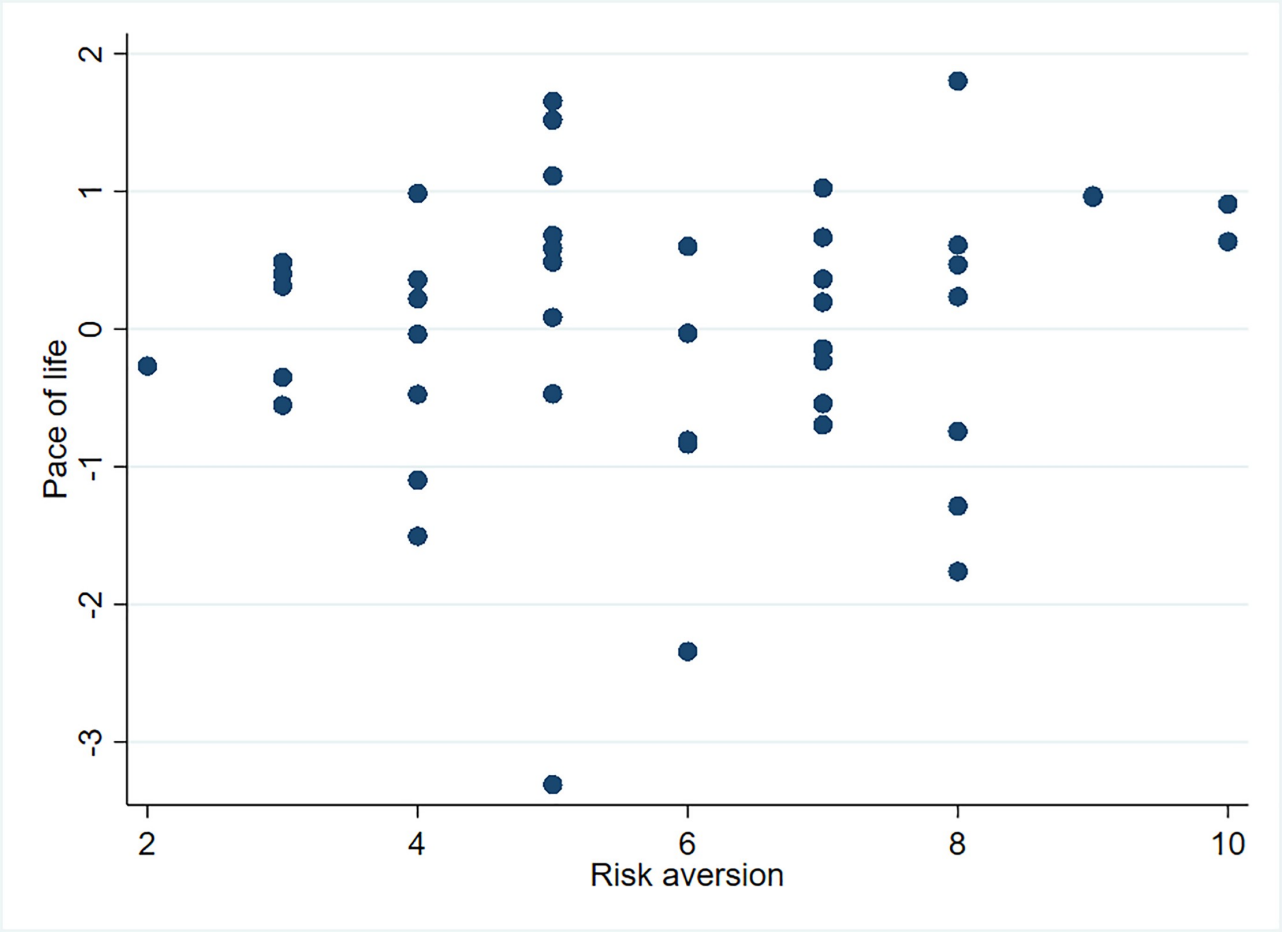
Pace of life and risk aversion.

**Fig 6:**
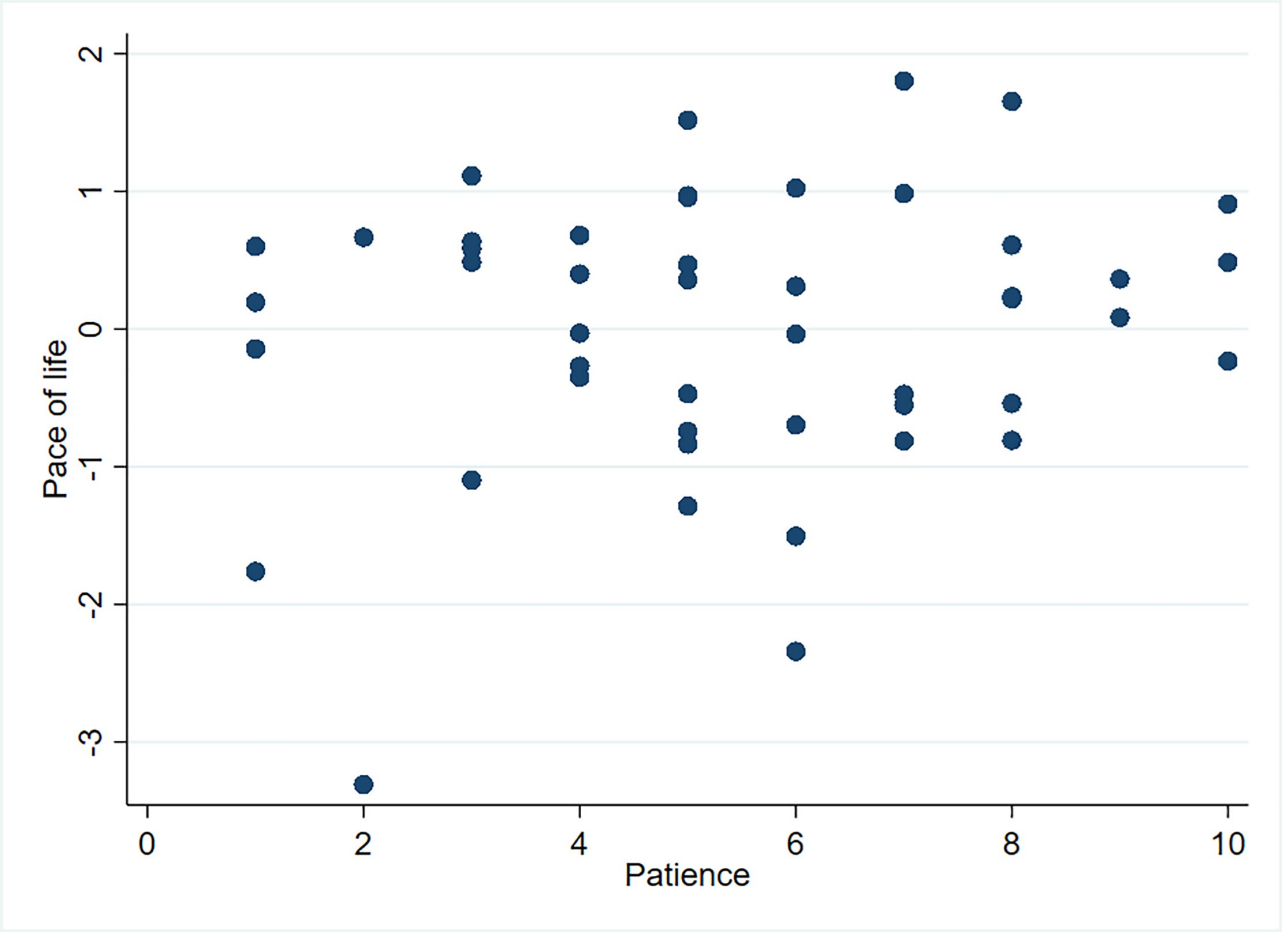
Pace of life and patience.

When observing the pace of life, patience, and risk (Figs 5 and 6), no strong tendencies can be detected. We found a small, positive, but insignificant correlation between the pace of life and risk and the pace of life and patience (see S3 Appendix).

Our results do not support hypothesis one, which postulates a strong correlation between the pace of life and risk attitude. However, risk attitude is still a relevant factor for explaining speed choice. We, therefore, include risk attitude in our later analysis.

### Pace of life and speed choice

For our multivariate analysis, with a binary dependent variable and longitudinal data, we run a random-effects probit model (see Table 4) We show that people who choose *fast* in the previous round are consistently more likely to choose *fast* in the next round. Results additionally show that people who had an accident in the previous round are very likely to choose *fast* in the next round. These results are consistent with those provided by [40] on the direct response mode's prevalence in route choice experiments. Direct responders are participants, who react proactively after facing a bad or no payoff in the previous round. Additionally, this behavior is predicted by gambler's fallacy, describing the belief that the probability of an event is reduced if it recently occurred (see, e.g., [41]). However, models (2) and (3) show that the probability of choosing *fast* is decreasing when the participant chose fast in the previous round and experienced an accident.

**Table 4:** Random-effects regression for choosing fast.

	(1)	(2)	(3)
Pace of life [0,1]			0.725*
			(0.386)
Fast (r-1)	0.715***	1.091***	1.090***
	(0.0437)	(0.0676)	(0.0676)
Accident (r-1)	0.225***	0.530***	0.530***
	(0.0426)	(0.0596)	(0.0596)
Fast (r-1) x Accident (r-1)		-0.633***	-0.632***
		(0.0856)	(0.0856)
Risk [0,1]	0.822***	0.792**	0.706**
	(0.307)	(0.309)	(0.300)
Patience [0,1]	0.0815	0.0926	0.00538
	(0.286)	(0.288)	(0.280)
Age	0.0124	0.0115	0.0221
	(0.0212)	(0.0213)	(0.0213)
Male [1=yes]	0.0338	0.0305	0.0376
	(0.154)	(0.155)	(0.149)
License [1=yes]	-0.296*	-0.296*	-0.354**
	(0.159)	(0.160)	(0.157)
Round (r)	-0.001	-0.001	-0.001
	(0.001)	(0.001	(0.001)
Constant	-0.721	-0.839	-0.839
	(0.648)	(0.652)	(0.652)
Observations	4,653	4,653	4,653
Number of independent observations	47	47	47
Log Likelihood	-2640.389	-2612.719	-2611.017
AIC	5300.777	5247.439	5246.034
BIC	5365.23	5318.337	5323.377

Standard errors in parentheses

*** p<0.01

** p<0.05

* p<0.1.

(r-1) = previous round.

Most importantly, we find a positive effect on the pace of life on driving fast, which is significant at the 10%-level. Thus, the probability of choosing fast is higher for fast-paced participants; see Table 4, model (3). This result supports our second hypothesis. Additionally, we show that risk-taking behavior has a significant positive effect on the decision to drive fast. Thus, risk-prone participants more often choose to drive fast. The results provided by the model (3) in Table 4 give crucial insights on the importance of the pace of life as a crucial personality measure, at least in the context of traffic situations. Expanding model (2) by the pace of life has only a very small reducing impact on the coefficient of the variable risk but results in a positive and significant coefficient for the variable pace of life. This result gives additional support for the rejection of hypotheses one and acceptance of hypotheses two. There is a significant negative effect on a license, which shows that participants who hold a driving license drive slower than those without. The participant's age, gender, and patience do not significantly affect choosing fast in our experiment.

Table 5 shows a Tobit model (see S4 Appendix for the density) for the cumulative variable of driving fast. Participants with a fast pace of life have a significant positive effect on driving fast, which supports the before-mentioned results in Table 4. Analyzing the results aggregated over all rounds, we see that the impact of the pace of life on the speeding decision seems to be even stronger than the risk measure, as the coefficient for risk becomes insignificant as far as the pace of life is included as explaining variable for choosing fast (see Table 5). Moreover, it is evident that participants who hold a driving license have a lower cumulative probability of driving fast.

**Table 5:** Tobit regression of the independent variable fast (cumulative).

	(1)	(2)
Pace of life [0,1]		16.26***
		(5.640)
Risk [0,1]	8.877*	6.945
	(4.655)	(4.331)
Patience [0,1]	-0.137	-2.086
	(4.342)	(4.049)
Age	-0.157	0.0806
	(0.326)	(0.311)
Male [1=Yes]	-1.378	-1.238
	(2.358)	(2.169)
License [1=Yes]	-5.229**	-6.513***
	(2.427)	(2.277)
Constant	31.19***	18.66*
	(9.931)	(10.12)
Number of independent observations	47	47
Log-Likelihood	-160.064	-156.242
AIC	334.129	328.484
BIC	347.080	343.285

Standard errors in parentheses

*** p<0.01

** p<0.05

* p<0.1

Our results support our idea that the pace of life and speeding are strongly related to each other and that the effect is independent of risk attitude.

### Pace of life and strategy change

The third hypothesis questions whether the pace of life affects the individual's inclination to change their strategy. One possible idea is that players characterized by a different pace of life also differ in their response to extreme results, i.e., changing their strategy from *fast* to *slow* or vice versa. Therefore, the dependent variable is a binary one that indicates whether the player changes his strategy from either *fast* to *slow* or vice versa (=1 player switches to the opposite strategy, =0 does not switch). Table 6 summarizes the interaction of life's pace with the occurrence of an accident or the fact that it has chosen *fast* in the previous round.

**Table 6:** Random-effects regression for strategy changes in speeding.

	(1)	(2)	(3)	(4)	(5)
Pace of life [0,1]	-0.824*** (0.310)	-0.835*** (0.315)	-0.804** (0.316)	-0.787** (0.323)	-0.760** (0.338)
Fast (r-1)			-0.106** (0.042)	-0.197*** (0.044)	
Accident (r-1)		0.317*** (0.0415)		0.365*** (0.043)	0.400*** (0.140)
Accident (r-1) x Pace of life [0,1]					-0.129 (0.208)
Risk [0,1]	-0.074 (0.240)	-0.077 (0.243)	-0.054 (0.244)	-0.036 (0.250)	-0.077 (0.244)
Patience [0,1]	0.002 (0.224)	-0.006 (0.228)	-0.006 (0.228)	-0.023 (0.234)	-0.008 (0.228)
Age	-0.016 (0.017)	-0.017 (0.017)	-0.016 (0.017)	-0.016 (0.018)	-0.017 (0.017)
Male [1=Yes]	0.067 (0.119)	0.085 (0.121)	0.070 (0.122)	0.091 (0.124)	0.085 (0.121)
License [1=Yes]	0.083 (0.126)	0.082 (0.128)	0.073 (0.128)	0.061 (0.131)	0.082 (0.128)
Round (r)	-0.002** (0.001)	-0.002*** (0.007)	-0.002*** (0.001)	-0.002***(0.001)	-0.002*** (0.001)
Constant	0.214 (0.558)	0.071 (0.567)	0.288 (0.568)	0.099 (0.581)	0.021 (0.574)
Observations	4,700	4,653	4,653	4,653	4,653
Number of id	47	47	47	47	47
Log-Likelihood	-2728.973	-2680.082	-2706.517	-2669.865	-2679.891
AIC	5475.946	5380.163	5433.033	5361.731	5381.781
BIC	5534.044	5444.616	5497.486	5432.629	5452.679

Standard errors in parentheses

*** p<0.01

** p<0.05

* p<0.1.

(r-1) = previous round.

We again run a random-effects probit model and find a significant negative and very robust effect between the pace of life and strategy changes. This highlights that the faster the participants' pace of life, the fewer their strategy changes. This result supports hypothesis 3. The significant negative effect of the pace of life on strategy changes is remaining nearly constant if we again control for driving fast and/or having an accident in the previous round. Having suffered from an accident in the previous round increases the probability of changing the strategy. Additionally, participants who chose fast in the previous round are also less likely to change their strategy. Together with the results obtained above (a higher pace of life increases the probability of choosing fast), these results support hypotheses 2 and 3.

Furthermore, we run a Tobit model to analyze the effect of the participants' characteristics on the total number of strategy changes. Table 7 highlights in the model (3) that the pace of life has a significant negative effect on the number of strategy changes. This illustrates that participants with a faster pace of life possess a lower number of strategy changes.

**Table 7:** Tobit regression on the number of strategy changes.

	(1)	(2)	(3)
Pace of life [0,1]			-27.81***
			(8.988)
Risk [0,1]	-5.218	-5.169	-1.978
	(7.574)	(7.541)	(6.911)
Patience [0,1]	-1.968	-2.052	1.239
	(7.070)	(7.040)	(6.468)
Age	-0.119	-0.159	-0.561
	(0.529)	(0.530)	(0.498)
Male [1=Yes]	3.347	4.166	3.879
	(3.822)	(3.986)	(3.615)
License [1=Yes]	0.287	0.0950	2.363
	(3.941)	(3.934)	(3.640)
Number of accidents		0.153	0.129
		(0.223)	(0.202)
Constant	29.90*	21.18	43.90**
	(16.12)	(20.44)	(19.93)
Log-Likelihood	-180.024	-179.788	-175.449
AIC	374.049	375.576	368.8982
BIC	387.000	390.3776	385.5495

Standard errors in parentheses.

*** p<0.01

** p<0.05

* p<0.1.

## Discussion and conclusion

Our paper contributes to the literature on traffic behavior by considering a factor that has sometimes been brought up in speeding discussions but is not systematically analyzed: Pace of life. Grounded in biological research, there is some evidence that an individual's pace of life affects human behavior. This study, therefore, set out to test several hypotheses. To do so and investigate the link between individual pace of life and risky speed choices in traffic, we collected several measures of the pace of life. We conducted a controlled laboratory traffic experiment that left participants with the choice between fast and slow to get from A to B. An advantage of obtaining speed decision data through a controlled and incentivized laboratory experiment is that it does not rely on self-reports which might suffer from social-desirability or recall biases and that repeated choices -and therefore changes in choices- can be observed. The first hypothesis (H1) predicts that the pace of life and risk might be correlated. However, we do not find a significant correlation. Unlike biological research, we do not measure risky behavior (e.g., [29,30]) but risk attitude. As there is a gap between attitude and behavior and obviously, attitude cannot be analyzed in animals, one explanation might be that the correlation only exists between risky behavior and pace of life, but not between risk attitude and pace of life.

This might also be the reason why risk attitude and pace of life individually and separately contribute to the explanation of choosing *fast* in the experiment. Our second hypothesis postulated that the pace of life and choosing the risky strategy *fast* are associated. More precisely, we assumed that the higher an individual's pace of life, the more often the individual chooses to drive fast. Our results support this hypothesis and are in line with biological research showing that animals and species with a higher pace of life are more likely to move faster, e.g., concerning tongue flicking behavior in snakes or showing higher aggressive behavior and activity level [1,32]. Individual differences in the perceptions of time were already found to be relevant in early work on speeding [2], and have recently re-introduced in the debate [19]. Our findings also stress that these inter-individual perceptions are highly relevant for choices. We argue that the psychological concept of pace of life [20,21] may be a relevant concept to predict risky decisions. Very recent research [31] also assumes a relation between pace of life and risk-taking.

Additionally, we also found support for the last hypothesis based on the observation that higher-paced snakes are more likely to repeat what they already did and are less likely to explore new areas [1]. This is reflected in an individual's inclination to strategy changes: We find that individuals with a higher pace of life are less likely to change their strategy, even after having experienced a loss. By considering both findings together, we find that high-paced individuals are more likely to make risky choices and are also likely to stick to once-established routines. Slow-paced individuals are more likely to explore different choices but are also more likely to choose the less risky alternative. This pattern is related to the ambidextrous relation of exploration-exploitation [42], which suggests that some individuals tend to rapidly exploit established paths while others are more likely to explore new strategies.

Overall, our results confirm that time perceptions – measured as the pace of life – contribute to our understanding of speeding decisions. Participants' behavior was assessed by several tasks, including a novel speeding experiment with monetary incentives instead of answering abstract questions in a questionnaire. Participants were confronted with a scenario where they were repeatedly asked to choose between driving fast or slow. Despite many methodological advantages, our experimental set up has some limitations, which could lead to possible future extensions. Firstly, experiencing an accident in the experiment has less severe implications and occurs more often than car crashes in real life and might be perceived differently. Furthermore, speeding participants have advantages over slow-driving participants, but experience the same negative outcome in case of a crash.

In future research, one could think about differently framed road risks and more substantial consequences, which might differ between fast and slow driving participants. Additionally, one could think about experimental outcomes that differ in the time spent in the experiment. So far, participants spend the same time engaged in the experiment, independent of whether they chose fast or slow. Future research could account for these technical limitations and improve the present findings that, nevertheless, add to the existing literature.

Our findings highlight that introducing the pace of life into behavioral models of speeding is needed next to future methodological advancements. Our experiment shows clear evidence that the individual's pace of life is associated with risky speeding decisions. The higher an individual's pace of life, the higher the likelihood to choose fast. Pace of life may explain not only inter-individual differences in speeding but also differences in speeding differences across countries. We leave this idea for future research. Our results are in line with recent biological literature on the pace of life, and our experiment is – up to our knowledge – the first experiment with humans to find a relation between habituation tendencies and pace of life. This association is particularly interesting as the tendency to explore new strategies and the tendency to stick to one chosen strategy is relevant for not only traffic decisions but in other situations, such as economic activities like entrepreneurship and strategic decisions.

## Supporting information

S1 Appendix (DOCX)

S2 Appendix (DOCX)

S3 Appendix (DOCX)

S4 Appendix (DOCX)
