# Supplementary material for: The fast and the furious—An experimental investigation of the pace of life and risky speed choice in traffic
Source: PLoS One. 2020 Jul 27;15(7):e0236589. doi: 10.1371/journal.pone.0236589 (PMC7384623; doi:10.1371/journal.pone.0236589)
Supplement: S1 Appendix — (DOCX) [file pone.0236589.s001.docx]

**Appendix S1**

**Instructions for the speed choice experiment**

In this experiment, **you and** **seven other participants** have to repeatedly travel from a **starting point A** to an **arrival point B** by car. All participants have the same origin and destination. The experiment consists of **100 periods.**

- You can either choose to travel fast (**F**) or slow (**S**) to get from A to B.
- For the choices the following holds:
  - If you drive fast and arrive in arrival point B, your payoff will be 2 experimental units.
  - If you drive slowly and arrive in arrival point B, your payoff will be 1 experimental unit.
  - Be aware: if you have an accident, you will not arrive in arrival point B and your payoff is 0 experimental units!

There won’t be accidents if all eight participants drive slowly. However, accidents can happen if one or more people drive fast. In general, the more people drive fast, the higher is a driver’s likelihood of having an accident. In that case, the likelihood of having an accident is higher for fast than for slow drivers.

- **Your information per period**:
  - Number of the current period
  - Your choice in the preceding period
  - An information whether you reached your destination with the last choice
  - Your period payoff in the preceding period in **experimental units**
  - Your cumulative payoffs in **experimental units**
- Independent of your success in the game, you will receive a **5 Euro** lump-sum payment for showing up and answering a survey after the game. The exchange rate is **7 Cent** per experimental unit.
