# Supplementary material for: The fast and the furious—An experimental investigation of the pace of life and risky speed choice in traffic
Source: PLoS One. 2020 Jul 27;15(7):e0236589. doi: 10.1371/journal.pone.0236589 (PMC7384623; doi:10.1371/journal.pone.0236589)
Supplement: S2 Appendix — (DOCX) [file pone.0236589.s002.docx]

**Appendix S2**


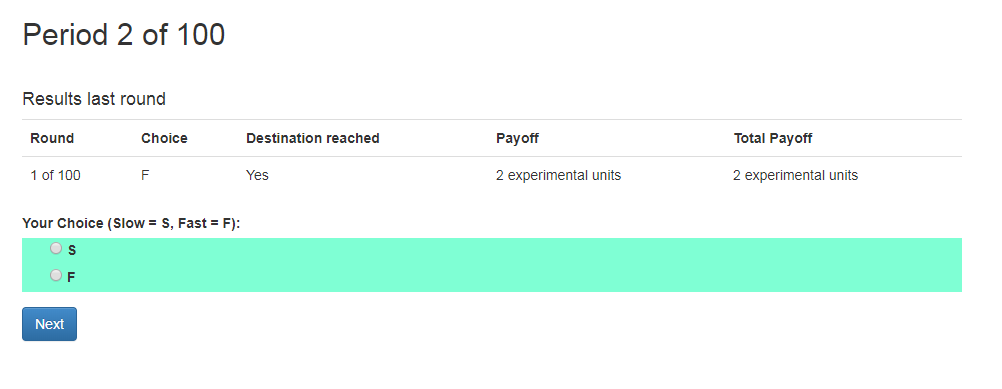


Fig S2: Screen-shot of the screen in the second round that shows the information participants received about the first round and asks them to choose the second round
