# Supplementary material for: The fast and the furious—An experimental investigation of the pace of life and risky speed choice in traffic
Source: PLoS One. 2020 Jul 27;15(7):e0236589. doi: 10.1371/journal.pone.0236589 (PMC7384623; doi:10.1371/journal.pone.0236589)
Supplement: S3 Appendix — (DOCX) [file pone.0236589.s003.docx]

**Appendix S3**

Table S3: Correlations

|  | Pace of life | Fast | Accumulated number of fast rounds per individual | Number of strategy changes | Accident | Risk | Patience | Age | Gender | License | Round |
| --- | --- | --- | --- | --- | --- | --- | --- | --- | --- | --- | --- |
| Pace of life | 1.0000 |  |  |  |  |  |  |  |  |  |  |
| Fast | 0.0770*** | 1.0000 |  |  |  |  |  |  |  |  |  |
| Accumulated number of fast rounds per individual | 0.1799 | 0.2412*** | 1.0000 |  |  |  |  |  |  |  |  |
| Number of strategy changes | -0.3819*** | 0.0005 | 0.0325 | 1.0000 |  |  |  |  |  |  |  |
| Accident | -0.0109 | 0.2386*** | 0.0281* | -0.0018 | 1.0000 |  |  |  |  |  |  |
| Risk | 0.1461 | 0.1548*** | 0.3619 | -0.0807 | -0.0140 | 1.0000 |  |  |  |  |  |
| Patience | 0.1912 | -0.0386*** | -0.0903 | -0.0133 | -0.0173 | 0.0146 | 1.0000 |  |  |  |  |
| Age | -0.1382 | -0.0116 | -0.0270 | -0.0341 | 0.0190 | -0.0850 | 0.1983 | 1.0000 |  |  |  |
| Gender | -0.0012 | 0.0626*** | 0.1454 | 0.1294 | -0.0574*** | 0.1337 | 0.1793 | -0.0546 | 1.0000 |  |  |
| License | 0.1277 | 0.1227*** | -0.2868** | -0.0101 | 0.0280* | -0.2182 | 0.1884 | 0.0612 | -0.2037 | 1.0000 |  |
| Round | 0 | -0.0099 | 0.7768*** | 0.7576*** | -0.0075 | 0 | 0 | 0 | 0 | 0 | 1.0000 |

*** p<0.01, ** p<0.05, * p<0.1
