# Supplementary material for: The fast and the furious—An experimental investigation of the pace of life and risky speed choice in traffic
Source: PLoS One. 2020 Jul 27;15(7):e0236589. doi: 10.1371/journal.pone.0236589 (PMC7384623; doi:10.1371/journal.pone.0236589)
Supplement: S4 Appendix — (DOCX) [file pone.0236589.s004.docx]

**Appendix S4**

Fig S4: Density of cumulative fast for the 100 rounds
